# High CO_2_ and Silicate Limitation Synergistically Increase the Toxicity of *Pseudo-nitzschia fraudulenta*

**DOI:** 10.1371/journal.pone.0032116

**Published:** 2012-02-21

**Authors:** Avery O. Tatters, Fei-Xue Fu, David A. Hutchins

**Affiliations:** Department of Biological Sciences, University of Southern California, Los Angeles, California, United States of America; University of New South Wales, Australia

## Abstract

Anthropogenic CO_2_ is progressively acidifying the ocean, but the responses of harmful algal bloom species that produce toxins that can bioaccumulate remain virtually unknown. The neurotoxin domoic acid is produced by the globally-distributed diatom genus *Pseudo-nitzschia*. This toxin is responsible for amnesic shellfish poisoning, which can result in illness or death in humans and regularly causes mass mortalities of marine mammals and birds. Domoic acid production by *Pseudo-nitzschia* cells is known to be regulated by nutrient availability, but potential interactions with increasing seawater CO_2_ concentrations are poorly understood. Here we present experiments measuring domoic acid production by acclimatized cultures of *Pseudo-nitzschia fraudulenta* that demonstrate a strong synergism between projected future CO_2_ levels (765 ppm) and silicate-limited growth, which greatly increases cellular toxicity relative to growth under modern atmospheric (360 ppm) or pre-industrial (200 ppm) CO_2_ conditions. Cellular Si∶C ratios decrease with increasing CO_2_, in a trend opposite to that seen for domoic acid production. The coastal California upwelling system where this species was isolated currently exhibits rapidly increasing levels of anthropogenic acidification, as well as widespread episodic silicate limitation of diatom growth. Our results suggest that the current ecosystem and human health impacts of toxic *Pseudo-nitzschia* blooms could be greatly exacerbated by future ocean acidification and ‘carbon fertilization’ of the coastal ocean.

## Introduction

The relentless consumption of fossil fuels is forcing carbon dioxide (CO_2_) into the oceans at the rate of ∼20–25 million tons day^−1^, driving a steady decrease in seawater pH often termed ocean acidification [1], [2]. Marine phytoplankton can be sensitive to the rising partial pressure of CO_2_ (pCO_2_) and lowered pH, as well as other concurrent environmental changes such as decreases in nutrient supplies to surface waters due to enhanced stratification [3]. Only of late, however, have oceanographers begun to examine how these ocean global change trends will affect the phytoplankton species that cause ecosystem-damaging toxic algal blooms [4]. Recent documented global increases in the frequency and severity of harmful algal blooms are hypothesized to be linked to changing anthropogenic atmospheric emissions and nutrient supplies, but direct causal evidence is lacking [5]. In particular, the possible synergistic effects of multiple climate change variables remain largely unexplored [3].

Among the most widely distributed and environmentally destructive harmful bloom species are diatoms of the genus *Pseudo-nitzschia* [6]. Many *Pseudo-nitzschia* species produce the potent toxin domoic acid, which binds with high affinity to vertebrate kainite-type ionotropic glutamate neuroreceptors [7]. Domoic acid is the agent of Amnesic Shellfish Poisoning, which causes illness or mortality in humans, marine mammals, and seabirds that ingest fish or shellfish that have been contaminated through bioaccumulation in coastal food webs [8], [9]. Previous studies have highlighted multiple triggers for increased domoic acid production, including nutrient limitation, extreme pH increases, trace metal availability and nitrogen source changes [10]–[16]. Recently Sun *et al*., (2011) notably demonstrated that culture toxicity increased substantially when phosphorous-limitation is combined with higher pCO_2_ in a *Pseudo-nitzschia multiseries* clone from Nova Scotia, Canada [17].

Extremely elevated levels of domoic acid have recently been reported from *Pseudo-nitzschia* blooms in coastal waters of Southern California [18]. One of the common species within these mixed blooms is *P. fraudulenta*. Our study examined how the availability of the required diatom nutrient silicate (Si(OH)_4_) interacts with changing pH and pCO_2_ (8.4 and 200 ppm pre-industrial; 8.2 and 360 ppm modern day; and 7.9 and 765 ppm, projected year 2100) to influence toxicity, growth, and silicon utilization in a *P. fraudulenta* clone isolated from Southern California waters.

## Results and Discussion

We found that the concentration of domoic acid in *P. fraudulenta* cells increases significantly under Si(OH)_4_ limitation, as has been reported in prior studies for other *Pseudo-nitzschia* species [10], [11], [19], [20]. Our results demonstrate, however, that this previously recognized Si(OH)_4_ effect on cellular toxin production rates is dramatically magnified (>250%) during growth at projected end-of-century atmospheric pCO_2_ levels (765 ppm). Thus, these Si(OH)_4_-limited diatom cells produce more than twice as much toxin when grown in acidified seawater. This nutrient/acidification synergism is environmentally relevant, as the California coast where this species was isolated is among the first ocean regimes where anthropogenically-acidified surface seawater has been documented during upwelling events [21]. Coincidentally, Si(OH)_4_ limitation of diatoms is also a common biogeochemical feature in parts of this regime [22].

**Figure 1:**
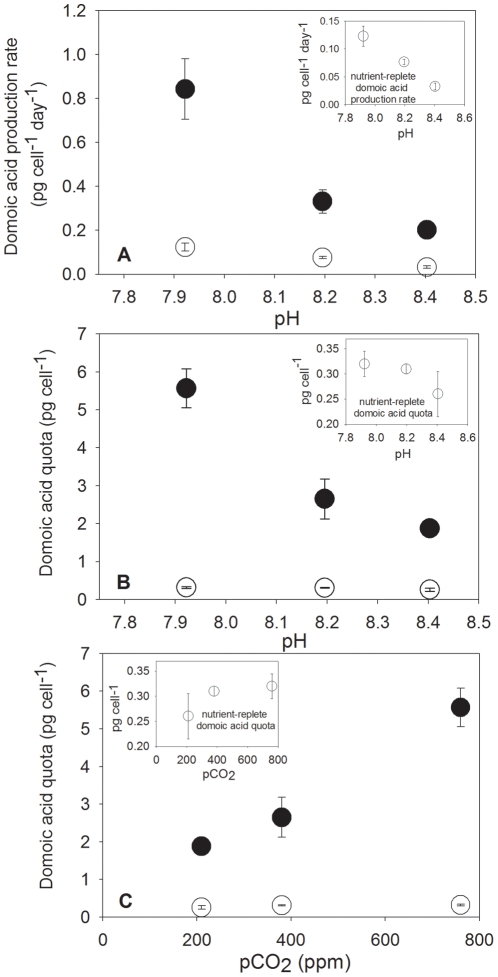
Interactive effects of pH and pCO_2_ with nutrient limitation control *Pseudo-nitzschia fraudulenta* toxicity. Cellular domoic acid production rates (pg cell^−1^ day^−1^) versus pH (**a**) and domoic acid quotas (pg cell^−1^) versus pH (**b**) and pCO_2_ (**c**) in *Pseudo-nitzschia fraudulenta* cultures grown under Si(OH)_4_-limited (•) and nutrient-replete (○) conditions at seawater CO_2_ concentrations of 200 ppm (preindustrial atmospheric levels), 360 ppm (modern levels), and 765 ppm (projected year 2100 levels). Panel insets present the nutrient-replete data with an expanded Y-axis scale for clarity. Error bars represent standard deviations of triplicates for each treatment.

This strong toxin production synergism between Si(OH)_4_ limitation of *Pseudo-nitzschia fraudulenta* and ocean acidification is shown in Fig. 1a. Relative to nutrient-replete cultures grown at the same pH, the cellular domoic acid production rates (pg cell^−1^ day^−1^) of Si(OH)_4_-limited diatom cells were elevated ∼4–7 fold (Fig. 1a, p<0.0001). Within these Si(OH)_4_-limited treatments, cellular domoic acid production rates were highest in cultures acclimated at projected year 2100 seawater pH (∼7.9). Toxin production rates within this acidified treatment were increased ∼4.2-fold compared to cells maintained at pre-industrial pH (∼8.4, p = 0.001), and 2.5-fold relative to those maintained at modern pH levels of ∼8.2 (Fig. 1a, p = 0.004). There was a highly significant negative linear correlation between domoic acid production rates and pH in these Si(OH)_4_-limited cultures (r^2^ = 0.94). Toxin production rates of nutrient-replete cells were substantially reduced, but also increased significantly (p<0.01) in a linear manner (r^2^ = 1.0) with successive decreases in pH (Fig. 1a, inset).

Similar to cellular production rates, cellular domoic acid quotas (total cellular toxin content, pg cell^−1^) were 7–18-fold higher in Si(OH)_4_-limited cultures relative to nutrient-replete cultures (Fig. 1b, p<0.0001). Domoic acid quotas in these Si(OH)_4_-limited treatments were 3-fold higher at pH 7.9 than at pH 8.4 (p = 0.0003), and increased 1.4-fold at pH 7.9 relative to pH 8.2 (p = 0.002). The inverse relationship between toxin quotas and pH under Si(OH)_4_-limitation was again highly linear (r^2^ = 0.94) across the three pH levels. Due to the inherent chemical relationships of the seawater carbonate buffer system, in the Si(OH)_4_-limited treatments cellular domoic acid quotas (and production rates, not shown) were also strongly positively correlated with pCO_2_ (r^2^ = 0.99, Fig. 1c). The much lower domoic acid content of nutrient-replete cells also increased at lower pH and higher pCO_2_, but these toxin quota changes were not significant (panel insets, Fig. 1b,c, p>0.05).

**Figure 2:**
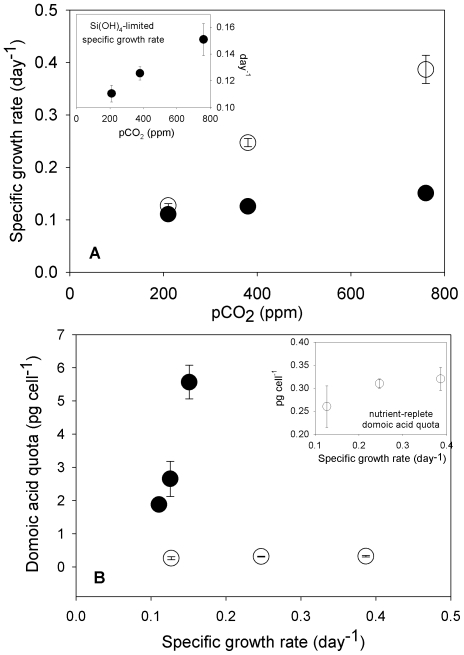
Relationships between nutrient-limited growth rates, pCO_2_, and toxicity in *Pseudo-nitzschia fraudulenta*. Specific growth rates (day^−1^) versus pCO_2_ (**a**) and cellular domoic acid quota (pg cell^−1^) versus specific growth rates (**b**) under Si(OH)_4_-limited (•) and nutrient-replete (○) conditions at three seawater CO_2_ concentrations (200, 360 and 765 ppm). Panel insets present the Si(OH)_4_-limited (**a**) or nutrient-replete (**b**) data with an expanded Y-axis scale for clarity. Error bars represent standard deviations of triplicates for each treatment.

**Figure 3:**
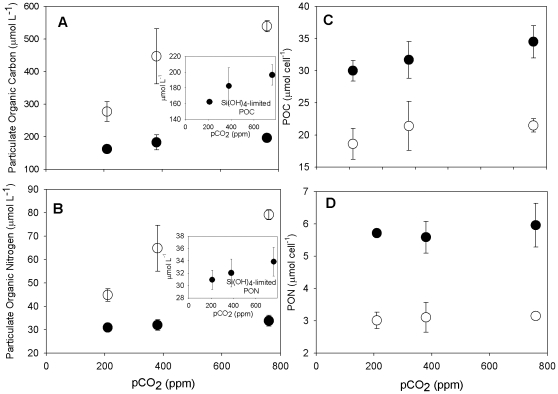
Steady-state particulate organic carbon (POC) (a) and nitrogen (PON) (b) concentrations (µmol L^−1^), particulate organic carbon (POC) (c) and nitrogen (PON) (d) cell quotas (mol cell^−1^) in semi-continuous *Pseudo-nitzschia fraudulenta* cultures grown under Si(OH)_4_-limited (black circles, •) and nutrient-replete (open circles, ○) conditions at three seawater CO_2_ concentrations (200, 360 and 765 ppm). Panel insets present the Si(OH)_4_-limited data with an expanded Y-axis scale for clarity. Error bars represent standard deviations of triplicates for each treatment.

As intended in our experimental design, specific growth rates of Si(OH)_4_-limited *P. fraudulenta* cultures were significantly reduced compared to nutrient-replete cells in all three pCO_2_ treatments (p<0.01, Fig. 2a). In the nutrient-replete cultures, there were significant progressive increases in growth rates across all three pCO_2_ levels, with increases of 48% and 66% relative to the 200 ppm treatment at 360 and 765 ppm, respectively (p<0.0009). Si(OH)_4_-limited culture growth rates also increased significantly with pCO_2_, but to a lesser degree (p<0.03, Fig. 2a, inset). These trends were confirmed in the semi-continuous cultures by steady-state concentrations of particulate organic carbon and nitrogen, proxies for total diatom biomass in the treatments (Fig. 3a,b).

The relationship between growth rate and cellular domoic acid quota was bimodal. Elevated domoic acid levels were only produced by cultures below a specific growth rate threshold of ∼0.15 d^−1^; at all higher growth rates in the nutrient-replete cultures, cellular toxin quotas were low, and there was no evident relationship between growth rate and cellular toxin content across the data set as a whole (Fig. 2b). However, due to the stimulatory effect of CO_2_ on both growth and toxicity, cellular domoic acid quotas were positively linearly correlated with growth rates within each nutrient treatment (Si(OH)_4_-limited, r^2^ = 0.99; nutrient-replete, r^2^ = 0.96, Fig. 2b and inset).

The trends we observed in toxin levels could be due in part to changes in individual cell mass or biovolume under our experimental treatments. Measurements of particulate organic carbon and nitrogen cell quotas (mol cell^−1^) suggested a 1.5 to 2-fold increase in cell size in all Si(OH)_4_-limited cultures relative to nutrient-replete treatments grown at the same pCO_2_ levels (Fig. 3c,d, p<0.0001), a not-uncommonly observed phenomenon in slowly growing, Si(OH)_4_-limited diatoms. Cell mass also increased slightly but not significantly with pCO_2_ (Fig. 3c,d, p>0.05). Thus the higher cell-normalized toxin levels in Si(OH)_4_-limited, high CO_2_-grown cells could be partially related to increases in the size and volume of individual *P. fraudulenta* cells. However, the observed increases in cellular toxin quotas in our experimental Si(OH)_4_-limited treatments (7–18-fold) were up to an order of magnitude greater than the corresponding increases in cell mass suggested by the data in Fig. 3c,d, so both nutrient limitation and ocean acidification appear to have physiological consequences resulting in large increases in toxin production per unit of diatom biomass.

**Figure 4:**
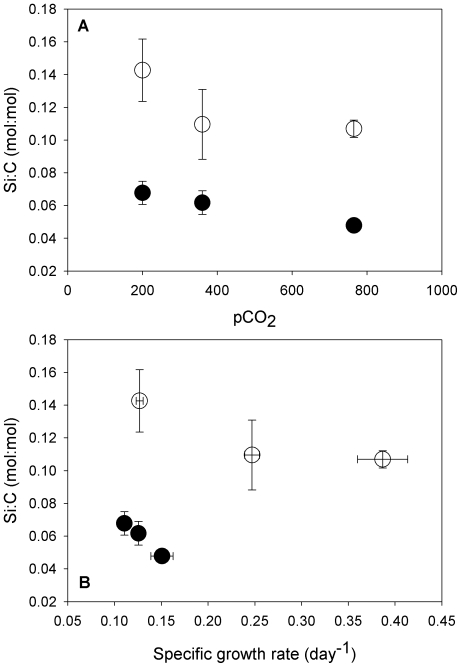
Relationships between cellular Si∶C ratios, pCO_2_, and growth rates in *Pseudo-nitzschia fraudulenta*. Cellular silica to particulate organic carbon (Si∶C, mol∶mol) ratios versus pCO_2_ (**a**) and cellular Si∶C versus specific growth rates (**b**) under Si(OH)_4_-limited (•) and nutrient-replete (○) conditions at three seawater CO_2_ concentrations (200, 360 and 765 ppm). Error bars represent standard deviations of triplicates for each treatment.

Cellular biogenic silica to particulate organic carbon (Si∶C) ratios were significantly lower at 765 than at 200 ppm ppm CO_2_ in both Si(OH)_4_ treatments (p<0.05), although neither was significantly different from Si∶C ratios at 360 ppm (p>0.05, Fig. 4a). These data are corroborated by the findings of Sun *et al*., (2011) in a *P. multiseries* clone grown at a similar range of pCO_2_ [17]. Milligan *et al*., (2004) observed lower Si cell quotas (mol Si cell^−1^) under more acidic conditions in cultures of the centric diatom *Thalassiosira weissflogii* due to higher cellular Si efflux∶influx ratios [23]. However, unlike the results of Milligan et al., cellular Si quotas in our *P. fraudulenta* isolate did not differ significantly between CO_2_ treatments (data not shown), and the Si∶C trends we observed were instead due entirely to increasing cellular particulate organic carbon quotas at higher CO_2_ concentrations (Fig. 3c). It is notable that in our pennate diatom, Si∶C increases at low CO_2_ occurred even in Si(OH)_4_-limited cultures (Fig. 4a).

Cellular Si∶C ratios were negatively linearly related to culture growth rates in both Si(OH)_4_-limited (r^2^ = 0.99) and Si(OH)_4_-replete (r^2^ = 0.77) cultures (Fig. 4b). This observation that Si∶C ratios are highest in diatoms under CO_2_-limited growth conditions (Fig. 4b) is similar to the Si∶C increases typically seen in diatoms growing under iron limitation [22], [24]. The inverse trends in cellular Si∶C ratios relative to CO_2_ levels and growth rates are however the opposite of the positive linear correlations we observed for toxin production rates and quotas versus these same two variables (Figs. 1c, 2b).

The synergism between elevated pCO_2_ and nutrient limitation in a widespread *Pseudo-nitzschia* species suggests that ocean acidification or ‘carbon fertilization’ could dramatically amplify the already considerable worldwide ecosystem impacts of toxic blooms, especially under commonly occurring nutrient-limited conditions. The west coast of North America where we isolated this clone is the first region where upwelling of anthropogenically-acidified seawater has been unambiguously demonstrated [21]. This same coastline is also plagued by large, frequently recurring toxic *Pseudo-nitzschia* blooms [18], [25], [26]. When fossil fuel-derived ocean acidification combines with naturally elevated pCO_2_ from upwelled deeper waters, surface water pH values here can be as low as 7.6 and pCO_2_ levels can reach 1000–1200 ppm [21]. By comparison, pH values of ∼7.9 and pCO_2_ of 765 ppm more than doubled diatom toxin production rates in our cultures, relative to modern atmospheric pCO_2_ levels (Fig. 1a).

In our experiments, CO_2_-driven increases in toxin production were much more evident in cultures limited by Si(OH)_4_ than in nutrient-replete treatments. Regulation of domoic acid production has been previously linked to Si(OH)_4_ limitation in several other *Pseudo-nitzschia* species [10], [11], [19], [20]. Inverse correlations between Si(OH)_4_ levels and domoic acid concentrations have been demonstrated during *Pseudo-nitzschia* blooms in southern California waters, and modeling approaches have suggested a role for Si(OH)_4_ limitation in determining domoic acid toxicity throughout much of central and southern California [18], [27], [28]. Chronic iron limitation of diatoms in some California upwelling centers also leads to ecosystem-level Si(OH)_4_-limitation, since iron-stressed cells produce heavier silica frustules and so deplete Si(OH)_4_ from surface waters long before other nutrients [22], [24].

The combination of high pCO_2_, acidified water with frequent and widespread Si(OH)_4_ limitation of toxic *Pseudo-nitzschia* assemblages could have major consequences for marine environmental health in the California region, and in other areas where similar conditions exist. Indeed, an environmental survey data set from the northern Gulf of Mexico shows correlations between low Si(OH)_4_ availability, elevated dissolved inorganic carbon concentrations, and increased toxin levels in *Pseudo-nitzschia* cells [29]. Our study is also validated by the findings of Sun *et al*., (2011), who found elevated domoic acid levels in another species (*P. multiseries*) cultured under phosphorous-limited, low pH (high pCO_2_) conditions [17]. However, our results may have considerably broader environmental implications since previous studies suggest that Si(OH)_4_-limitation of *Pseudo-nitzschia* growth is likely to be much more common and widespread than phosphorous-limitation [18], [27], [28].

In addition to increased toxicity, increasing pCO_2_ resulted in significantly higher specific growth rates for both nutrient-replete and Si(OH)_4_-limited cells (Fig. 2a), as well as increased carbon fixation rates (data not shown). Thus, ocean acidification could not only enhance the toxicity of *Pseudo-nitzschia* blooms, but may also increase growth rates and biomass, even under Si(OH)_4_-limited conditions when cellular domoic acid production is elevated.

The physiological and genetic mechanisms underlying this CO_2_-nutrient limitation toxicity synergism are undetermined at present, but may have to await elucidation of the full biosynthetic pathway of domoic acid. Complete synthesis results from condensation of an alpha-ketoglutarate derivative stemming from the tricarboxylic acid cycle with a C-10 isoprenoid unit likely originating from an unknown compartmentalized pathway within the cell. As this latter component of the synthetic pathway is poorly understood [30], our ability to obtain a truly mechanistic picture of how domoic acid production is regulated by environmental variables is limited. Application of modern transcriptomic and proteomic methods to toxic *Pseudo-nitzschia* cultures of varying potencies is likely to be productive in this regard; the ability to quantitatively and sensitively up- and down-regulate cellular domoic acid levels by manipulating the nutrient conditions and pCO_2_ at which cultures are grown may prove invaluable for such mechanistic molecular studies. Future work also needs to examine how the interactions between rising pCO_2_ and other global change trends such as warming temperatures and increased irradiance in shallower ocean mixed layers [3], [4] will affect *Pseudo-nitzschia* growth and toxicity. In particular, increasing pCO_2_ combined with iron limitation, which was also demonstrated to regulate domoic acid production [14], [15], [31], should be considered in future studies, as well as co-limitation by multiple factors including iron, light, silicate, and nitrate [32].

The implications of our results are not limited to the distant future. In fact, the range of pCO_2_ levels found in anthropogenically-acidified waters of the California upwelling regime today already encompasses those used in our study, including projected average year 2100 atmospheric concentrations [21]. Thus, *Pseudo-nitzschia* bloom toxicity may already be affected by changing pCO_2_ levels here, and this area may be considered a natural laboratory in which to evaluate the effects of future global ocean acidification on harmful algal blooms. Strong synergistic effects of rising pCO_2_ with other variables such as nutrient limitation emphasize the need for heightened future vigilance by marine environmental regulatory and protection agencies, as well as a possible need to add new parameters such as seawater carbonate system measurements to existing harmful algal bloom detection and monitoring efforts. Such unprecedented measures may well be necessary to help mitigate escalating environmental and economic damage from increasingly toxic *Pseudo-nitzschia* blooms in a rapidly acidifying ocean.

## Materials and Methods

### Culture, Media, and Sampling

*Pseudo-nitzschia fraudulenta* was micropipette isolated from public nearshore water collected at 34.08 N, 119.05 W in Ventura County, California in March, 2010. This isolate, designated USC WWA7, was maintained at 16°C on a 12-h light: 12-h dark cycle in modified f/2 enriched seawater growth media [33] under 90 photons m^−2^ s^−1^ of cool white fluorescent illumination. Silicate (Si(OH)_4_) final concentrations in the medium were 10.6 µM for the Si(OH)_4_-limited treatment and 106.1 µM for the nutrient-replete treatment; these concentrations were chosen based on previous experience with cultures of *Chaetoceros* and *Thalassiosira* spp. Dissolved CO_2_ concentrations were controlled by gentle bubbling with commercially prepared air/CO_2_ mixtures (Praxair Gas) at approximately 120 bubbles·min^−1^ with in-line HEPA filters to avoid particulate contamination.

### Cell Counts and Growth Rates

Steady state semi-continuous culture methodology was employed to maintain cultures in exponential state and specific growth rates were calculated as described in Sun *et al*., (2011) [17]. Growth rate as determined by bi-weekly microscopic cell counts and *in vivo* fluorescence determined the dilution rate of each bottle. Cells of *P. fraudulenta* preserved in acidified Lugol's solution were vortexed and enumerated by direct counts using an Accu-Scope 3032 inverted microscope according to the Utermöhl method [34]. A minimum of 300 cells were counted to guarantee a 95% confidence interval with +/−11.5% accuracy [35]. Cultures were acclimated for a period of three months to the respective experimental conditions prior to splitting into triplicates, which were then further acclimated for one month (5–17 cell divisions) prior to final sampling.

### Domoic acid by high performance liquid chromatography

High performance liquid chromatography with ultraviolet detection (HPLC-UV) of domoic acid was performed using a SCL-10ADVP controlled system (Shimadzu). The UV detector was programmed for sample and reference wavelengths of 242 and 280 nm respectively and the system was operated by EZ START software version 7.4 SP1 (Shimadzu).

Cellular concentrations of domoic acid were determined according to Mafra et al., (2009) with slight modifications [36]. Culture subsamples of 10–20 ml were carefully measured and cells were collected by gentle filtration on 25 mm GF/F filters (Whatman). The filters were stored in the dark at −20°C. Filters were subsequently subject to sonication in 10% aqueous methanol for 2 min. at 40W in a water bath. The cell extracts were then clarified by centrifugation at 3000× *g* for 10 min at 4°C. The pellet was discarded and the clarified extracts were transferred to 300 µl polyspring inserts (National Scientific) placed inside clean 2.0 ml Target DP™ vials (National Scientific). Prior to analysis, all samples were treated with 0.15% trifluoroacetic acid (TFA).

Briefly, the chromatographic separation was carried out on a reversed phase Luna C18 (2) column (3 µm, 2×100 mm, Phenomenex) at 25° C with a mobile phase system consisting of water with 0.1% TFA (A), and acetonitrile (MeCN) with 0.1% TFA (B). The elution gradient began with a 10–35% B transition over 10 min, then was held at 35% B until 15 min, followed by a subsequent decrease to 10% B at 16 min, and held at 10% B. The flow rate was 0.2 ml min^−1^ and the injection volume 5–10 µl. Quantification of domoic acid was determined using certified reference material CRM-domoic acid-e obtained from the National Resource Council, Canada at a range of concentrations.

Calibration curves of CRM-domoic acid-e were determined by linear regressions (r^2^ values≥0.99) for each sample treatment. Interpolation from the standard curves was used to calculate the amount of compound injected from the peak areas of each sample under the same experimental conditions. Domoic acid per cell concentrations were determined by the ratio of reconstituted volume (300 µl) to the volume injected (5–10 µl) and dividing by the total cell count in the original sample. Reported values represent means of the results (n = 3). Domoic acid production rates were calculated by multiplying the growth rate by toxin per cell.

### Carbonate Buffer System analysis

Dissolved inorganic carbon analysis was performed using a CM5230 CO_2_ coulometer (UIC) [17]. Samples were transferred carefully without agitation into 25 ml liquid scintillation vials fitted with Teflon (PFTE) coated caps, poisoned with 200 µl of 5% HgCl_2_ l^−1^, and stored at 4°C until analysis. pH was determined on freshly collected samples using a calibrated Orion 5-star plus pH meter using an NBS buffer system with three-point calibration. The pCO_2_ in the experimental media was calculated from these two parameters using CO_2_SYS software [37]. Measured NBS scale pH values averaged across all the replicates in the three treatments were 8.43, 8.23, and 7.95; measured total dissolved inorganic carbon concentrations were 1965, 2107, and 2249 µmol kg^−1^; and the corresponding calculated pCO_2_ values were 198, 357, and 764 ppm. For clarity, pCO_2_ treatments in the cultures are referred to in the text using rounded-off values of 200 ppm, pre-industrial atmospheric levels; 360 ppm, modern atmospheric levels: and 765 ppm, projected year 2100 levels [38].

### Chlorophyll *a*

For chlorophyll *a* (Chl *a*) measurements, samples were filtered in duplicate onto 25 mm GF/F filters. Five ml of 90% acetone was later added and each vial was allowed to extract overnight in the dark at −20°C. After twenty-four hours, Chl *a* was determined using a Turner Designs 10-AU fluorometer [39].

### Particulate organic carbon and nitrogen

For particulate organic carbon (POC) and nitrogen (PON), sample volumes of 20 ml were collected onto pre-combusted (450°C for 5 h) GF/F glass fiber filters, stored at −20°C, and dried at 55°C before analysis. Molar POC and PON content was analyzed using a 4010 Costech Elemental Combustion System calibrated with methionine and atropine as reference materials according to the methods in Hutchins *et al*., (1998) and Fu *et al*., (2007) [40], [41].

### Biogenic Silica

Sample volumes of 20 ml were collected onto 0.6 µM polycarbonate filters, dried and stored at ambient temperature until analysis. Cellular biogenic silica (BSi) was measured according to Brzezinski and Nelson (1995) [42].

### Statistics

Differences between treatments for all parameters were tested using two-way ANOVA and the individual treatments were compared using Tukey's Multiple Comparison Test or Student's t-test. All analyses were performed using SigmaPlot 10 software.
